# Integrated Digital Patient Education at the Bedside for Patients with Chronic Conditions: Observational Study

**DOI:** 10.2196/22947

**Published:** 2020-12-22

**Authors:** Benjamin Schooley, Akanksha Singh, Neşet Hikmet, Robert Brookshire, Nitin Patel

**Affiliations:** 1 Health Information Technology Program College of Engineering and Computing University of South Carolina Columbia, SC United States; 2 Department of Computer Science and Engineering College of Engineering and Computing University of South Carolina Columbia, SC United States; 3 Department of Medicine School of Medicine University of South Carolina Columbia, SC United States; 4 Digital Health Prisma Health Columbia, SC United States

**Keywords:** patient education, patient understanding, blended learning, adherence, digital patient education, chronic condition, understanding, outcome, mHealth, education, digital health

## Abstract

**Background:**

Patient education delivered by a health care provider increases patients’ understanding and adherence to medical instructions, which helps to improve patient health. Multiple challenges exist to delivering effective patient education to patients with multiple chronic conditions, including giving the necessary time, range, and types of learning materials, and assessing the level of understanding. To help overcome these challenges, it is important to study new electronic means to assist in patient education, such as the use of mobile devices, interactive media, 3-dimensional images, and multimedia educational content at the bedside.

**Objective:**

The goal of this study was to address the need for blended learning strategies combining technical and workflow integration of digital patient education systems for patients with chronic conditions within and across the regular process of care. Studies are needed to evaluate the utility and benefits of these technologies for providers and patients alike.

**Methods:**

A mixed-methods approach was employed including survey administration to 178 patients after they received digital patient education in person with a health care provider, and qualitative interviews with 16 nurse educators who used the mobile digital health education technology to deliver instruction to patients. Patient survey data were analyzed using chi-square statistical tests. Qualitative interviews were analyzed for user acceptance and perceived value themes.

**Results:**

Patients who were counseled using a blended digital health education approach reported improved understanding of educational content (*P*=.034) and chronic health conditions (*P*<.001), were more motivated to care for themselves at home (*P*<.001), were more likely to say that they felt capable of making health care decisions with their doctors (*P*<.001) and on their own (*P*=.001), and were more likely to report their intention to follow their doctor’s instructions (*P*<.001) than were patients whose education was not computer-based. Nurse educators felt that the digital education system and content enhanced their education efforts and could be easily integrated into the outpatient clinical workflow.

**Conclusions:**

Patient education for individuals with chronic conditions may be more effective than traditional formats when provided in blended digital formats supervised by a health care provider.

## Introduction

### Background

Patient education increases patient understanding and adherence to medical instructions, which has a major impact on long-range outcomes for patients with chronic health conditions [1-3]. A recent meta-analysis of 320 articles on patient compliance with instructions and its associated impacts on physiological progress and long-range health outcomes for chronic disease management found that patient education was the most successful of all experimental effects [4]. Information technology (IT) facilitates fundamental changes to patient education delivery, enabling digital media content that is considered a time-effective and cost-effective alternative to traditional patient education [5]. The use of digital technologies for patient learning has increased drastically through the application of web-based patient education sites, video and 3-dimensional (3D) multimedia content, mobile devices (such as smartphones, MP3 players, iPods [Apple Inc], and tablets), and other devices and technology media [6]. However, many questions remain about which digital educational delivery strategies are best for a given setting.

Patients with chronic conditions require a holistic approach for care, education, and self-management to achieve patient understanding and motivation to adhere to instructions [7]. Lack of patient awareness about a condition has been identified as the most common barrier to active self-management of chronic conditions [8]. Further, despite good patient understanding about a specific treatment, adherence worsens with increasing age and complexity of treatments [9]. For patients with multiple comorbidities, there is a greater need for in-depth education that includes provider interaction [7,10,11]. Indeed, improving patient education for people with chronic conditions may be an important component to improving individual and community health delivery systems, decision making, and related outcomes [12]. Providing better education with the level of personalization that health care providers are able to deliver is an approach advocated by patients with chronic conditions [13].

Prior research has validated the use of a wide range of education and communication interventions for improving patient comprehension [14]. For example, some educational modes include using cartoon images [15], educational booklets [16], educational pamphlets [17], simple visual aids [18], and telephone-based education and reinforcement [19]. These education modes have been shown to have a positive effect on patients. Positive effects have also been demonstrated with digital and computer-enhanced education, including educational videos to supplement a traditional office consultation, web-based education programs that utilize text and voice reminders, digital multimedia and 3D interactive content [3,20], animated messaging, personalized video and flash content based on patient health status and demographic characteristics [21], tablet/PC-based education in place of nurse-led conventional patient education [22], use of multimedia presentations [21], mobile apps [22], and a wide range of other digital tools across various health care and patient contexts [23,24]. These studies have largely demonstrated improved patient education as well as higher satisfaction levels among providers [25]. Results have also indicated high levels of technology acceptance for the purpose of patient education, while also indicating that solutions need to be tailored to the needs of providers and specific patient populations [17-20].

Prior digital patient education studies have been conducted in patients undergoing or recovering from specific procedures, such as total joint arthroplasty and total hip replacement, endovascular aneurysm repair, peripheral angioplasty, catheter insertion procedures, endoscopy, and perioperative interventions, as well as in patients seeking to improve health conditions, including immunosuppression, oral health care, heart disease, lower back pain, asthma, and other conditions. It has been widely concluded that patients in specific medical contexts, receiving relevant content, have improved understanding as well as motivation to adhere to instructions when using digital learning formats, which in turn has led to higher levels of satisfaction among these patients [21,22,26-34]. Computer-aided learning has been found to have positive impacts on the knowledge, attitude, behavior, and health of patients [27].

Evaluation measures used to assess the effectiveness of computer-enhanced patient education in most studies include patient understanding, patient satisfaction, adherence to process, and improved usability. Younger, higher educated, and internet-savvy patients are found to be more tech savvy and have higher effectiveness levels when evaluated using these measures [5,35-38]. Patients have found the use of multimedia presentations during the consent process to be helpful in their understanding and report improved satisfaction. Increased regular use of multimedia presentations to help patients understand their prescribed procedures is found to be beneficial in the care of patients undergoing some interventions, including vascular and endoscopic procedures [34,35]. One study discovered that mobile device education apps support increases in adherence and patient satisfaction and help facilitate perioperative interventions [37]. Increased utilization of digital formats has shown to enhance patient education and related factors for remote, distance, online, or unsupervised patient education [39], and chronic disease management [40]. Indeed, patient education for chronic disease management is of paramount concern, as the number of individuals being diagnosed with a chronic condition in North America continues to increase, resulting in increased health care utilization [41-43].

Although there is no one-size-fits-all patient education approach, effective instruction may apply a variety of teaching tools, methodologies, and strategies that can be tailored to the individual patient’s circumstances and competencies, the learning environment, and the range and types of teaching tools available to providers [44]. Common strategies today include in-person and face-to-face instruction [45] (ranging in size from one person to large groups) and digital learning, where either patients or providers control the amount and type of learning [46]. In-person education is generally believed to allow for more effective question-and-answer communications between patients and providers and better visual and auditory determination of learning competency by the provider. Health care providers involved in the development of e-learning techniques suggest several strategies, including (1) tailoring information according to patient characteristics, (2) structuring information to be relevant and easy to find, and (3) being aware of and sensitive to patients’ emotions.

For online patient education in chronic disease management settings, essential design features include patient-tailored information, interactivity, content credibility, clear presentation of content, use of multimedia, and interpretability [34]. Moreover, e-learning is likely most effective when it facilitates individual learning needs, supports feedback on competence level and improvement, and allows input from significant others (eg, experts, peers, or patients) [36]. It is widely believed that achieving all these objectives can be very challenging when applying digital education on its own without health care provider interaction or intervention, especially for patients experiencing multiple conditions and comorbidities.

### Goal of the Study

This study investigated a blended learning patient education program that integrates technology-enhanced instruction with conventional in-person teaching approaches in the process of care [47]. This approach benefits from patient interactions with their provider and from mobile, digital, multimedia-enhanced education [48]. This blended learning approach for patients with multiple chronic conditions has not been extensively researched. One recent meta-analysis included just 12 studies that ranged in length from 4 weeks to 8 months and included both self-paced learning and face-to-face learning components [49]. This study focused on clinician-directed and facilitated digital education during one critical interaction in the regular course of care—a setting that represents the most common and opportune educational environment. The study evaluated differences between groups receiving traditional in-person education and in-person digital education. We evaluated differences in patient understanding, motivation for self-care, intention to adhere to instructions, confidence in care decision making, and satisfaction with clinician-patient communication by using digital technologies in the presence of a clinician in the regular flow of a patient visit (bedside). We employed digital technology in the presence of a nurse educator (NE) to augment and personalize traditional patient education methods in an outpatient clinical setting in the context of educating patients with chronic medical conditions.

## Methods

### Study Design

A mixed-methods approach was used to collect survey responses from patients receiving patient education and to evaluate NE perspectives on their use of the digital patient education system. The mixed-methods design used for this study follows the guidance of Morse and Niehaus [50], employing quantitative analysis of patient surveys and qualitative interviews and responses from NEs. Informed consent was obtained from all individual participants involved in the study in compliance with the approved University of South Carolina Institutional Review Board protocol (#Pro00059265) for this study.

### Overview

From October 2017 to May 2018, participants were recruited from the normal flow of patients visiting one large internal medicine clinic and one hospital in the southeastern United States. Patient participants were enrolled in the study at the time that they met with their doctor, after their doctor determined that they required patient education. Doctors relied on their clinical experience to assess patient education needs. Doctors generally selected patients for instruction who fit into at least one of the following categories: (1) very recently diagnosed with a new chronic condition, (2) new to the clinic with a diagnosed chronic condition, (3) not demonstrating compliance with medical instructions, or (4) not demonstrating expected health improvements after reportedly following medical instructions and prescriptions. Patients were eligible to participate if they were aged 18 years or older, required patient education as determined by their physician, were considered “complex” patients (ie, that they were being treated for more than one chronic condition), and deemed capable of completing a survey on their own without assistance. Physicians chose patients to enroll who were over the age of 45 years—with no patients over the age of 76 years (mean age 58 years)—who were being treated for two or more of the following types of conditions: diabetes, hypertension, congestive heart failure/heart disease, obesity, chronic obstructive pulmonary disease, anxiety/depression, stroke, kidney and/or bladder failure/disease, asthma, lung disease, arthritis, chronic back pain, or osteoporosis.

After providing their informed consent, patient participants were enrolled by the NEs into one of two study arms: (1) mobile device–enabled electronic multimedia education (intervention) or (2) usual paper/pamphlet-based education (control). The NEs, all of whom worked full-time for the participating health care institution, made every effort to alternate the assignment of patients into the two study arms. Nevertheless, this was an observational cohort study in which the assignment of the intervention was not fully at the discretion of the investigator [51,52]. A total of 16 NEs (11 ambulatory, 2 inpatient, and 3 both ambulatory and inpatient), including all of the NEs at the internal medicine clinic and the hospital inpatient educators affiliated with the same clinic, provided education in one of two formats. Patient activation measures were not collected for reporting. From a clinical standpoint, all patients in this study went through the standard patient activation process at the clinic. Participants assigned to the control arm received education face-to-face with an NE in the usual format that included paper handouts, brochures, printable content from the electronic health record, paper charts, and plastic models (eg, heart, skull, etc). The intervention group also received education face-to-face with an NE but using a touchscreen laptop computer with interactive 3D images, videos, graphs, and charts. In both groups, NEs were free to choose the content based on the diagnoses, care plan, and instructions given to each patient by his/her physician. NEs used educational material in topical areas related to internal medicine, health education, general surgery, oncology, cardiology, and orthopedics.

NEs were provided with 2 hours of training on the digital patient education tool and touchscreen laptop computer prior to patient enrolment. NEs were primarily instructed on how to use the technology and were not trained on the specific educational content. This is because all of the NEs in this study had already become proficient in providing education on all the topics noted above while using paper/pamphlet-based education. The same NEs were interviewed in person at the end of the study, after all patient surveys were completed. Researchers informed the NEs about the study and gained their consent to participate as per the approved Institutional Review Board protocol. Qualitative results are presented using guidance from the consolidated criteria for reporting qualitative research [53].

### Survey Data

An online survey was sent to each study participant at the conclusion of each patient education session. Patients responded to questions on demographics, patient understanding, patient-provider communications, motivation to care for self at home, intention to adhere to instructions, and patient confidence in care decision making on one’s own and together with a physician. A total of 178 patients completed the patient consent, face-to-face education, and survey instrument. The list of survey questions that were administered are provided in Textbox 1.

**Survey questions.**

Survey questions

- Do you understand your condition better now since receiving your education today?
- Did this educator’s use of a computer or handheld device make it harder or easier for you to talk with him or her?
- Did the instruction you received motivate you to care for yourself at home?
- Did the instruction you received help you feel capable of making health care decisions together with your doctor?
- Did the instruction you received help you feel capable of making your own health care decisions?
- As a result of the education you received, how likely are you to follow the instructions given to you by your doctor?

### Interview Data

All 16 NEs that provided standard and digitally enhanced education were interviewed for 30 minutes each in a private room at their workplace. Semistructured questions were asked that focused on the use of the technology, the benefits and challenges of using it in the process of patient care, and the perceived benefits and challenges to patients. Two researchers (BS and NP) conducted all of the interviews. The interviews were recorded and transcribed and analyzed using the Atlas.ti [54] qualitative analysis tool for emerging themes using a grounded theory approach [55]. Three researchers (BS, NP, and NH) worked together as a committee to code responses and modify coding until consensus was reached across all three. It was determined that data saturation was met after 13 interviews. NEs were primarily women (n=16) with a mean age of 36 years. All efforts were taken to protect the identity of participants and thus additional details are not provided. Participants were provided with the opportunity to review the study’s findings prior to publication.

## Results

### Survey Findings

Participants were asked to evaluate the effectiveness of the education received by responding to a series of questions on a 3-point Likert scale (“Yes, definitely;” “Yes, somewhat;” or “No”). A 3-point Likert scale was chosen for this study because it was quick to use by participants [56], with a low cognitive load. This was important as the survey was administered during the process of care in a busy outpatient clinic. Further, 3-point scales have demonstrated high reliability and validity and have been used effectively [57,58] in observational studies in health care [59,60]. Very few participants responded “No” to any of the questions, indicating that most participants found the education at least somewhat effective. However, there were significant differences between the group that received computer-assisted education and the group that received paper-based education in the number of responses of “Yes, definitely” as opposed to “Yes, somewhat.” The chi-square statistic was used to understand differences between observed counts. Participants’ demographics are shown in Table 1.

**Table 1:** Participants’ demographics (N=178).

Demographics		Values, n (%)
**Gender**		
	Male	77 (43.3)
	Female	101 (56.7)
**Ethnicity**		
	Black or African American	99 (55.6)
	White	77 (43.3)
	Hispanic or Latino	2 (1.1)
**Self-rating of health status**		
	Poor	6 (3.4)
	Fair	65 (36.5)
	Good	86 (48.3)
	Very good	17 (9.6)
	Excellent	4 (2.2)
**Level of education**		
	Less than high school	9 (5.1)
	High school graduate	57 (32.0)
	Some college or 2-year degree	45 (25.3)
	Four-year college graduate	52 (29.2)
	Graduate or professional education	15 (8.4)

Participants who were counseled using the tablet device and 3D/video-based education were more likely to report that the instructions they received were definitely easy to understand (109/115, 94.8%) compared with those who received paper materials (53/62, 85.5%; *P*=.034). Those receiving tablet-assisted counseling were much more likely to say they definitely understood their chronic health conditions better (99/116, 85.3%) compared with those who did not receive this type of counseling (36/61, 59.0%; *P*<.001). Similarly, those who were counseled with the tablet were much more likely to say that they were definitely motivated to care for themselves at home (90/116, 77.6%) than were those with paper-based materials (30/62, 48.4%; *P*<.001).

With regard to making health care decisions, participants who received counseling with the tablet were much more likely to say that they definitely felt capable of making health care decisions with their doctors (91/116, 78.4%) compared with those who received paper-based materials (27/62, 43.5%; *P*<.001), and they were also more likely to say that they definitely felt more capable of making their own health care decisions (90/116, 77.6%) compared with participants who received the paper-based materials (31/62, 50.0%; *P*=.001).

Finally, participants who received computer-based education said that they were very likely to follow their doctor’s instructions (92/116, 79.3%) compared with those whose education was not computer-based (25/62, 40.3%; *P*<.001).

### Interview Findings

Nurse educators (NEs) provided interview responses regarding the benefits and challenges of using the blended learning digital educational intervention compared with traditional paper-based education. Several themes emerged that were derived from the data. First, the NEs noted that the technology was easy enough to integrate into practice workflow. NEs gave comments such as, “It [the laptop and digital content] really wasn’t more difficult to use than my normal materials [paper/pamphlet learning material]” [NE5]; “It was pretty easy to find what I needed when I needed it” [NE11]; and “Using it didn’t slow me down” [NE1]. Second, patients seemed to pay better attention to 3D and video content. NEs said, “They [the patients] watch [the videos] pretty closely” [NE15]; “They [patients] like the pictures [interactive 3D], how they move around. They get interested, they actually ask questions” [NE7]; and “I’d say they pay better attention to the screen” [NE1]. Third, the system facilitates dynamic and efficient changes to and personalization of digital content delivery. Comments included, “I could make it [instruction] more about the patient” [NE9]; and “I like that I could give him [the patient] what they needed” [NE11]. Fourth, the system prompts on-demand question and answer sessions with patients. For example, one NE said, “It was pretty easy to just look up another video on a subject, like when a patient asked about surgery when I’m talking about [congestive heart failure], I could just have them watch the other video” [NE9]. Fifth, technology enables more easily accessible content for a broader range of educational categories to ensure that patients received education on topics that align with physician-directed patient instructions and prescriptions. NEs responded, “They [patients] could ask a question and there’s tons of pictures to click on to help answer it” [NE2]; and “I think it was a little easier to really look at what [the doctor] wanted for the patient to have, and then find it” [NE8]. Sixth, technology supports education under time- and resource-constrained circumstances. NEs stated, “By the time I’m sitting with the patient, they’re tired and ready to go home. They already saw the doctor, so they’re like, ‘It’s time to go!’ So it helps to get it [education] done sooner” [NE15]; “I see patients back to back, so not really time to go looking for a handout, or printing one” [NE3]; and “Yeah, I like having it all in the laptop. It’s just faster” [NE7].

NEs believed that the blended learning educational strategy also provided some advantages over online-only patient education. Themes that emerged included the following: (1) digital content prompts discussion, while in-person meeting allows for resolving concerns and misunderstandings by the patient and/or caregiver; (2) digital content ensures that the patient is receiving (watching/listening/reading) the educational content that pertains to his/her instructions and prescriptions; and (3) digital content allows the NE to experience verbal and visual cues to help assess patient understanding. Participating NEs explained, “There’s a lot of back and forth” [NE1]; “Pictures [3D anatomy pictures], those I could move, zoom in, rotate around and the patient is like, ‘cool’ and starts asking questions” [NE5]; “When they [patients] ask questions I know they’re listening” [NE10]; “Yeah, well they want to go home at that point, but they want to know, they’re just wanting to understand what they have to do. They talk [to me]” [NE11]; and “We’ll talk about their prescription and I can tell they’re learning about it. It’s like ‘that’s what my heart looks like?’” [NE9]. NEs noted several challenges with the blended learning strategy over traditional paper-based education. First, additional time and work can be required to ensure good, valid, and time-appropriate digital content. One NE commented, “There’s so much that I don’t need. I really just need to know where to go to get what I need, when I need it, and it took time to figure that out” [NE13]. Second, backup paper-based education is required because technology is not always working properly. For example, a few NEs explained, “The laptop updated, and I didn’t have time to wait [to give instruction]” [NE3]; and “It [computer] works pretty good. But I always have my handouts with me cause what if it [computer] doesn’t work. Sometimes it’s [computer] just not working for me” [NE7].

Several minor themes that emerged included that agreement is needed between educators on the most effective navigation tool to access content—depending on practice specialty and educational content needed (ie, there isn’t a one-size-fits-all efficient content structure). Comments included, “I had to learn where everything was” [NE14], referring to the placement of content; and “I wanted to show the stuff about stroke; it wasn’t where it should be” [NE9]. Second, significant bandwidth requirements are needed for accessing online/server-side digital media (eg, interactive 3D). NEs made comments such as, “It ran really slow sometimes” [NE2]; and “The videos were ok, but the pictures [3D images] could get really choppy” [NE8]. Finally, additional time and worry is required to work with the IT department for mobile technology security adherence. NEs explained, “I learned not to forget my password!” [NE11]; and “It took two hours for him [IT staff] to make it [the computer] work on the network” [NE9].

## Discussion

### Principal Findings

Education delivered by a health care provider to a patient is essential for increasing patient understanding and adherence to medical instructions and to assist with increasing the overall health of the patient [2,61,62]. While there is no unified theory on patient education delivery, how the education is delivered makes a difference in how it is received. While electronic means to assist in patient education continue to advance, studies are needed to evaluate the utility and benefits of these technologies for providers and patients alike [63], in a variety of health care settings. This study represents a comparative evaluation of patient education in two settings: traditional paper-based education, and digital content with tablet/PC-assisted in-person instruction, both in the presence of a clinician to assist in the education process for patients with multiple chronic conditions. The in-person nature of education provided in this study was chosen due to the known complexities of providing education to patients with multiple chronic conditions. Digital education provided to patients outside of the care facility (eg, at home) is often not consumed by the patient in part or in whole [64,65]. Thus, for this study providers were able to maintain a desired level of control over patient education delivery. Several past studies have focused on delivering patient education content using mobile devices with multimedia digital formats for the patient to view at home or unsupervised in a health care environment. This study addresses the need for technical and workflow integration of these systems within and across the regular process of care provision in person with the patient.

We evaluated differences in patient understanding, motivation for self-care, intention to adhere to instructions, confidence in care decision making, and satisfaction with clinician-patient communication by using digital technologies in the presence of a clinician in the regular flow of a patient visit (ie, bedside). Results indicate that the interactive and 3D content, delivered using a touchscreen tablet or PC, was perceived to be more impactful to patients than the traditional paper- and pamphlet-based education that NEs typically provide to patients. That is, patients who received the blended learning approach responded that they understood their condition better, were more motivated to care for themselves at home, felt more capable of making health care decisions both on their own and with their doctor, and reported that they were more likely to follow instructions given to them by their doctor compared with patients who received traditional education. These findings provide important insights for those who are tasked with designing patient education programs. As patient engagement has become an important pursuit across all health care organizations to increase compliance with medical instructions for value-based care, these findings demonstrate that the blended learning model, delivered through the regular course of a patient visit for chronic disease management, may provide improvements upon traditional education delivery. Studies have concluded that population subgroups with chronic conditions, who incidentally comprise a large portion of the population with multiple comorbidities, are less likely to follow programs that provide strictly digital health–only education. Rather, these patients may need more personalized and in-person education for chronic conditions management [65]. The NEs in this study likewise reported positive perceptions about integrating the digital education technologies into their workflow.

Behavior change is noted as the primary social determinant of value-based self-management of chronic conditions [12]. Remote patient education is growing with the use of patient portals used remotely, but the challenge is to efficiently and accurately measure patient understanding and patient adherence with the use of these portals. Moreover, health care providers have personal experiences and skills in patient education that are extremely useful in ensuring that patients understand the most important aspects of self-care with chronic conditions. Thus, provider-directed personalized education may be most favorable for achieving maximum patient understanding and motivation to adhere to instructions for patients with multiple chronic conditions.

In this study, a cloud-based, mobile, web-based patient education software system was utilized with patients face-to-face with NEs in an outpatient clinic. Our study is unique in at least two ways. First, we sought to deliver—and pilot—a cloud-based, mobile, web-based patient education application and mobile tablet computer within the context of bedside care for patients with multiple chronic conditions. While other studies have shown that media-enhanced instruction alone is at least as beneficial as standards-based nurse counseling [66], this study combined both strategies for a blended learning approach for the purpose of chronic disease management. Second, our study compared traditional paper/pamphlet-based education with multimedia and video-based instruction via a mobile device in the hands of NEs, providing personalized education guided by physician instructions and prescriptions in the normal process of a patient visit. The NEs were allowed the flexibility to tailor education as needed, based upon the knowledge level and questions of each patient, as well as patients’ individual diagnoses, prescriptions, and discharge instructions.

### Limitations

This study has certain limitations in its applicability. The assignment of the intervention was not at the discretion of the investigator and thus the study was not a randomized controlled trial [51,52]. NEs made every effort to alternate between using the intervention and using traditional paper-based educational materials for every other patient [51,52]. This effort to alternate the assignment of patients resulted in two groups with different group sizes. However, we believe that this should not be a limiting factor in our analysis. We also did not control for specific diagnoses or treatments; all of the subjects were considered “complex” patients, as defined by their physicians, and presented with chronic and/or multiple conditions. The sample size of the patient group was somewhat small and was also limited to one geographic area. We recommend future studies for evaluating patients with specific illnesses using a larger sample size.

### Future Directions

Comparative studies are needed to evaluate alternatives to human facilitators in patient education. Future studies may seek to investigate how to integrate personalized digital patient education, such as what was delivered in this study, for remote patient settings, such as using synchronous two-way video education for patients and providers to communicate. Future studies should consider measuring patient activation as a way to understand how blended patient education strategies affect the knowledge, skills, beliefs, and behaviors that a patient needs to manage a chronic illness.

### Conclusions

The outcome measures that were evaluated are patient understanding, patient motivation for self-care, patient confidence in care decision making, clinician-patient communication, and patient’s intention to follow instructions. Our results indicate that utilizing digital methods for patient education in the presence of a clinician to assist in the process is quite effective in enhancing these outcomes. We noted significant differences between the two groups evaluated. A blended learning patient education approach that integrates mobile 3D/video technology–enhanced instruction with conventional in-person teaching at bedside may be one important component of a comprehensive patient education strategy for chronic care management. Assessing study outcomes by providers who deliver the training along with patients who receive the education provide a balanced assessment framework for digital patient education.

## Abbreviations

IT: information technology
NE: nurse educator
3D: 3-dimensional
